# A molecular optomechanics approach reveals functional relevance of force transduction across talin and desmoplakin

**DOI:** 10.1126/sciadv.adg3347

**Published:** 2023-06-21

**Authors:** Tanmay Sadhanasatish, Katharina Augustin, Lukas Windgasse, Anna Chrostek-Grashoff, Matthias Rief, Carsten Grashoff

**Affiliations:** ^1^ University of Münster, Institute of Integrative Cell Biology and Physiology, Münster D-48149, Germany.; ^2^ Center for Protein Assemblies and Department of Bioscience, School of Natural Sciences, Technical University of Munich, 85748 Garching, Germany.

## Abstract

Many mechanobiological processes that govern development and tissue homeostasis are regulated on the level of individual molecular linkages, and a number of proteins experiencing piconewton-scale forces in cells have been identified. However, under which conditions these force-bearing linkages become critical for a given mechanobiological process is often still unclear. Here, we established an approach to revealing the mechanical function of intracellular molecules using molecular optomechanics. When applied to the integrin activator talin, the technique provides direct evidence that its role as a mechanical linker is indispensable for the maintenance of cell-matrix adhesions and overall cell integrity. Applying the technique to desmoplakin shows that mechanical engagement of desmosomes to intermediate filaments is expendable under homeostatic conditions yet strictly required for preserving cell-cell adhesion under stress. These results reveal a central role of talin and desmoplakin as mechanical linkers in cell adhesion structures and demonstrate that molecular optomechanics is a powerful tool to investigate the molecular details of mechanobiological processes.

## Teaser

Approach to control force-bearing linkages with light reveals molecular insights into cell-matrix and cell-cell adhesion.

## INTRODUCTION

Cells have the inherent ability to sense and respond to mechanical stimuli, and it is broadly recognized that the underlying mechanobiological mechanisms are central for a wide range of developmental, homeostatic, and pathological processes (*1*, *2*). Since many mechanical signals are transduced across macromolecular structures, which can contain hundreds of different proteins, the molecular details governing cellular mechanotransduction remain difficult to elucidate. Therefore, we and others have developed force sensing techniques to visualize and quantify where, when, and to what extent distinct molecules experience mechanical stress in cells (*3*, *4*). The tension sensor approach, for instance, uses single-molecule calibrated Förster resonance energy transfer**–**based biosensors to determine piconewton-scale forces inside cells (*5*–*8*), while recombinant or DNA-based tools are especially useful to study extracellular mechanics at the outer plasma membrane (*9*–*11*). These methods have helped to identify a growing list of mechanosensitive proteins that bear piconewton forces in cells and entire tissues (*12*–*15*). However, it often remained difficult to ascertain whether the force-bearing function of a molecule is relevant to the investigated cellular process. Given the redundancy of many mechanobiological networks and macromolecular structures (*16*, *17*), a molecular linkage experiencing mechanical forces may be dispensable, and its loss is quickly compensated by other load-bearing molecules. As a result, understanding the mechanobiology of cells on molecular scales has continued to be challenging.

In this study, we sought to establish an optomechanical (OM) approach that enables a specific and rapid breaking of molecular linkages with light to expose their functional relevance. Unlike a previously described method (*18*), we wanted to ensure that mechanical linkages form in the absence of light stimulation allowing normal cell behaviour, while light pulses are used to break the molecular connection with high spatiotemporal specificity. In contrast to methods based on chemical compounds (*19*) and photocleavable proteins (*20*, *21*), we aimed at a technique that is reversible, enabling cellular recovery and thus repetitive measurements. Furthermore, we wanted to avoid the use of ultraviolet (UV) irradiation to minimize phototoxic effects and permit extended live cell experiments.

We demonstrate the feasibility of this approach by applying the technique to the ubiquitously expressed protein talin, which physically connects integrin receptors with the actin cytoskeleton in complex, macromolecular structures called focal adhesions (FAs) (*6*, *7*, *22*). Light modulation in living cells provides direct evidence that talin does not only play a fundamental role as an integrin activator, as is widely recognized (*23*, *24*), but is also indispensable as a mechanical linker protein and critically required for maintaining cell adhesion integrity, despite the presence of other potentially force-bearing molecules in FAs. Implementation of the technique to examine the molecular mechanics of desmosomes (*25*) reveals that the desmoplakin (Dsp) linkage is dispensable for cell-cell adhesion maintenance under homeostatic conditions yet specifically required for intercellular adhesion integrity under conditions of externally applied mechanical stress.

## RESULTS

### Single-molecule force spectroscopy characterization of an optomechanical dimer

**Fig. 1.**
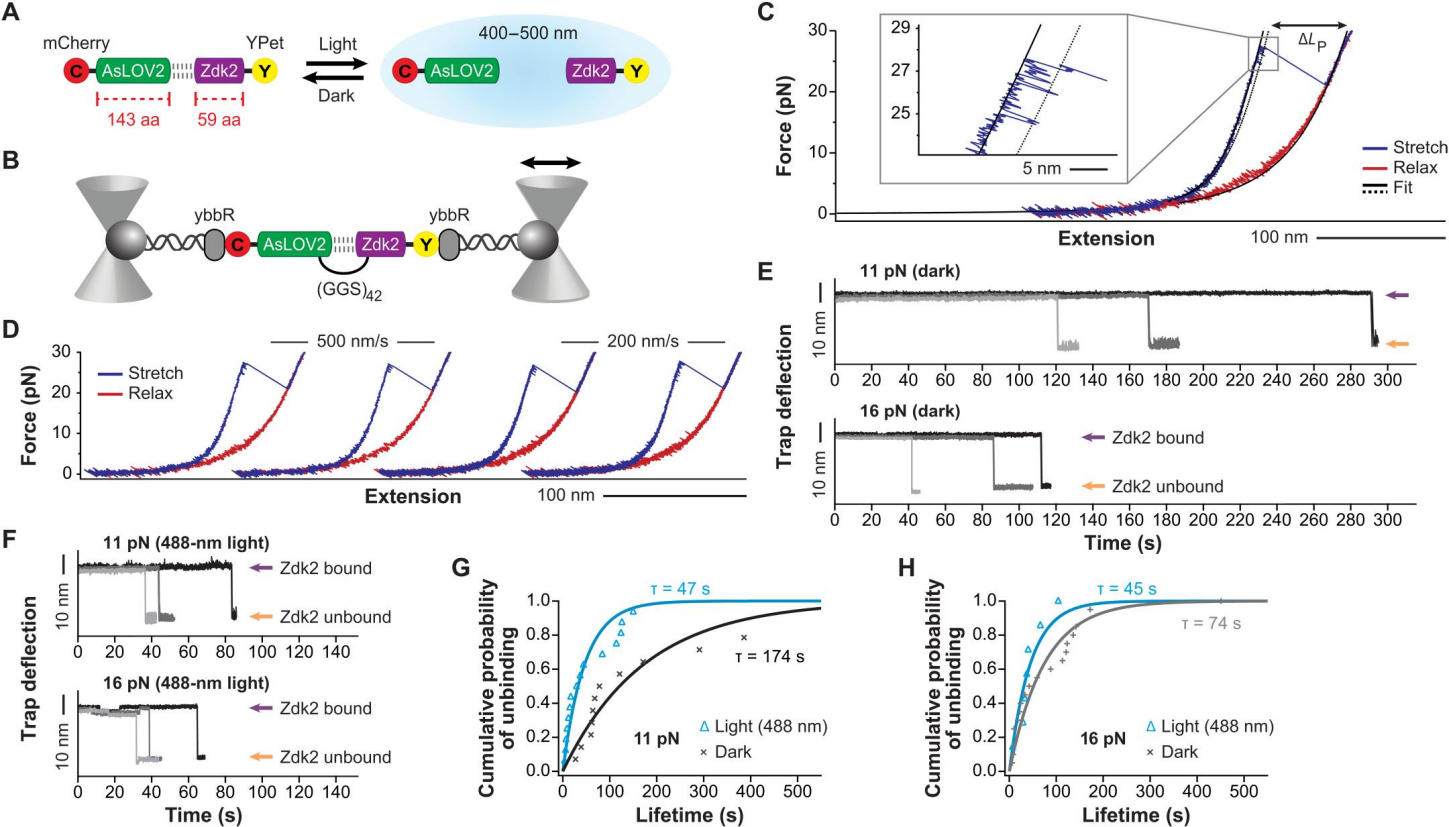
The AsLOV2-Zdk2 dimer withstands physiologically relevant forces. (**A**) Scheme of the AsLOV2-Zdk2–based molecular optomechanics module in its bound and unbound state. aa, amino acid. (**B**) Schematic representation of the optical tweezer experiments to measure single-molecule resolved unbinding forces. Application of mechanical force leads to repeated dissociation and rebinding events. (**C**) Representative force-extension curve of an AsLOV2-Zdk2 dimer. The zoom-in shows the characteristic flickering that is observed before rupture. Unbinding of Zdk2 occurs at around 24 pN leading to a contour length gain of Δ*L*_P_ = 70 nm (straight line, fit to DNA and to the peptide unbound state; dashed line, fit to partial unfolding region). (**D**) Series of repeated force-extension traces performed on a single AsLOV2-Zdk2 pair (blue, stretch; red, relax) at two pulling velocities (500 and 200 nm/s). (**E**) Representative traces of constant force experiments at 11 and 16 pN, under dark conditions. In each case, three traces are overlaid. (**F**) Representative traces of constant force experiments at 11 and 16 pN, in the presence of 488-nm light. Lifetimes at 11 and 16 pN are shortened and nearly identical. (**G** and **H**) Cumulative probabilities of unbinding events over time at 11 pN (G) and 16 pN (H) of load with and without illumination at 488 nm. Average lifetimes at 11 pN are 174 s without light (*n* = 14) and 47 s with light (*n* = 16). Average lifetimes at 16 pN are 74 s without light (*n* = 20) and 45 s with light (*n* = 7). Data were fit by a single exponential function.

The strategy described here uses a fluorescently tagged optogenetic dimer that stably connects two halves of a load-bearing molecule but can be rapidly yet reversibly dissociated using light stimulation (Fig. 1A). We hypothesized that a previously identified light-sensitive module, AsLOV2-Zdk2, may fulfil these requirements (*26*). However, it was unclear whether the dimer would resist physiologically relevant forces in the dark allowing the genetic engineering of intracellular, load-bearing linkages.

To test whether AsLOV2-Zdk2 is capable of withstanding mechanical forces at sufficiently high mechanical loads, we used a single-molecule force spectroscopy using dual-beam optical tweezers (*6*, *7*). We generated an expression construct in which AsLOV2 and Zdk2 were connected by a 126–amino acid–long, flexible linker peptide (GGS)_42_ to allow repeated measurements. In addition, we fused fluorescent proteins (mCherry and YPet) for later visualization of AsLOV2 and Zdk2 in cells and attached terminal ybbR tags for the association of DNA handles and the subsequent connection to microbeads (Fig. 1B). Single-molecule characterization of the resulting [ybbR-mCherry-AsLOV2-(GGS)_42_-Zdk2-YPet-ybbR] construct revealed that AsLOV2 and Zdk2 unbind at comparably high forces of about 24 pN, when a gradually increasing mechanical load was applied across the molecule (Fig. 1C and fig. S1A). Before the major Zdk2 unbinding peak characterized by a contour length increase (Δ*L*_P_) of about 70 nm, we observed a rapid transition between the folded and a partially unfolded region of the AsLOV2 (Fig. 1C, zoom-in). The characteristic unbinding at forces above 20 pN was highly reproducible and unaffected by different pulling velocities of 200 and 500 nm/s (Fig. 1D); the unbinding of AsLOV2 and Zdk2 always occurred before the unfolding of fluorophores that became evident at forces above 30 pN (*6*).

Molecular linkages are expected to withstand mechanical loads for tens of seconds in cells. Since our previous tension sensor experiments showed that individual molecules can experience forces of about 11 pN (*6*, *27*), we exposed individual linkages to a constant load of 11 and 16 pN and determined how long mechanical forces are borne before unbinding. On average, AsLOV2-Zdk2 unbinding occurred at forces of 11 pN after approximately 174 s and at 16 pN after about 74 s (Fig. 1, E to H). In 30% of experiments at 11 pN, the dimer did not dissociate even after a maximum waiting time of 9 min. However, exposure of the sample to 488-nm light strongly decreased the lifetime of the bound state under constant force, nearly fourfold at 11 pN and by about half at 16 pN (Fig. 1, F to H). Thus, in the absence of light, the AsLOV2-Zdk2 bond can withstand physiologically relevant piconewton-scale forces that are typically transduced across intracellular linkages.

### Engineering a light-sensitive talin junction in cells

**Fig. 2.**
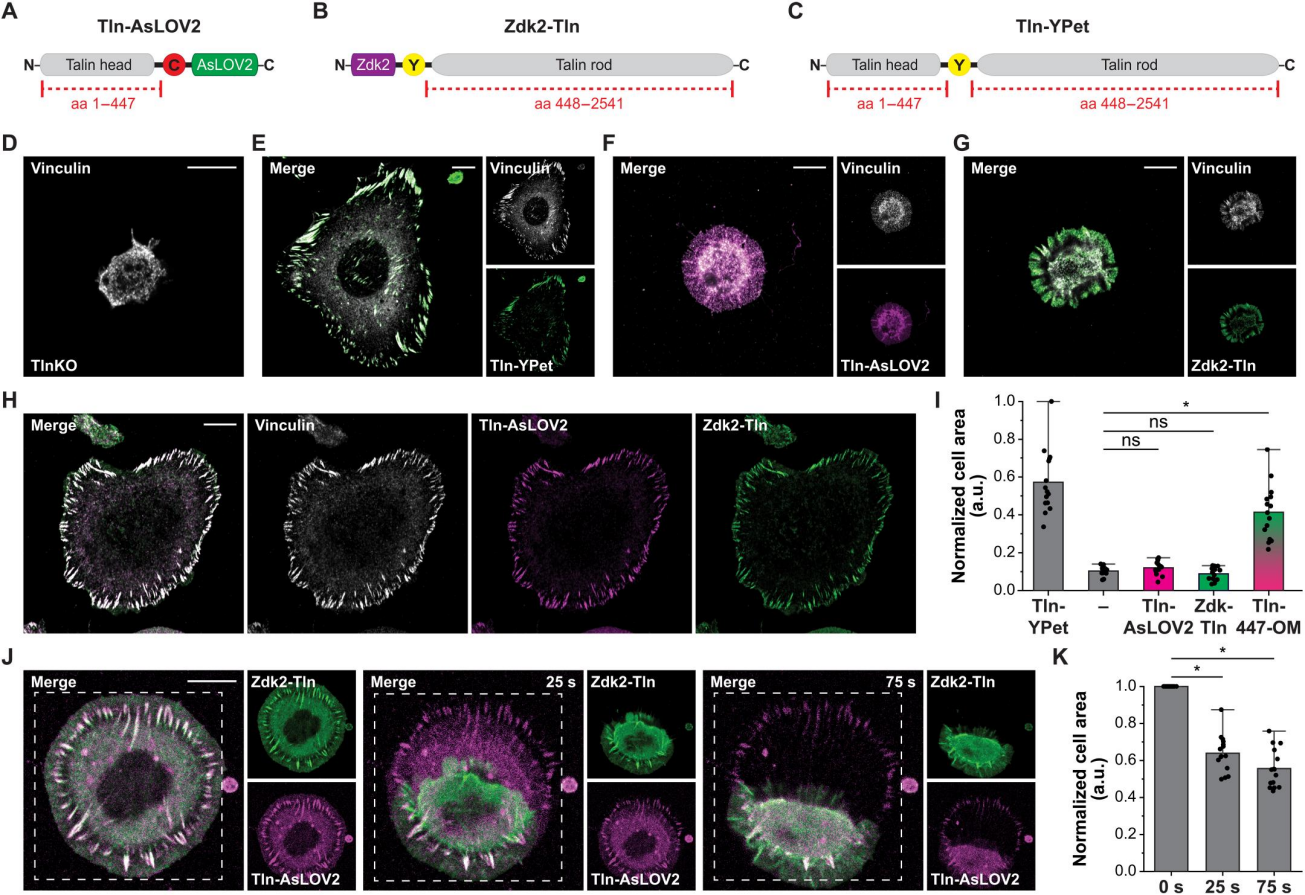
Engineering of light-sensitive, force bearing linkages. (**A** to **C**) Scheme of talin expression constructs showing the talin-1 head (amino acids 1 to 447) tagged with mCherry-AsLOV2 (Tln-AsLOV2) (A), the talin-1 rod (amino acids 448 to 2541) fused to Zdk2-YPet (Zdk2-Tln) (**B**), and talin-1 internally tagged with YPet (Tln-YPet) (C). (**D** to **H**) Representative images of a vinculin-stained TlnKO cell (Tln1^−/−^Tln2^−/−^) (D) and TlnKO cells expressing Tln-YPet (E), Tln-AsLOV2 (F), Zdk2-Tln (G), or coexpressing Tln-AsLOV2 and Zdk2-Tln (H). (**I**) Quantification of the normalized cell area reveals that the coexpression of Tln-AsLOV2 and Zdk2-Tln (Tln-447-OM) can restore cell spreading in TlnKO cells (*n* = 15; Mann-Whitney *U* test). a.u., arbitrary units. (**J**) Image series of a living TlnKO cell coexpressing Tln-AsLOV2 (magenta) and Zdk2-Tln (green) before and under light stimulation with 458 nm. The irradiation area is indicated by the dashed line. Note the rapid separation of mCherry (magenta) and YPet (green) signals upon stimulation. (**K**) Quantification of normalized cell area before, 25 s, and 75 s after the start of light stimulation (*n* = 15; paired sample Wilcoxon signed-rank test). Scale bars, 10 μm. *P < 0.05; ns (not significant), *P* ≥ 0.05.

To evaluate whether the AsLOV2-Zdk2 dimer can be used in cells to generate light-sensitive force-bearing linkages, we targeted talin-1 that connects the β subunit of integrin receptors with the actin cytoskeleton (*22*). It is established that talin-1 is an essential integrin activator and therefore indispensable in all adherent cell types (*28*). Talin also experiences mechanical forces upon cell adhesion formation (*6*, *7*, *29*, *30*), and a previous OM study revealed that mechanical engagement of the C-terminal part of the talin-rod domain is required for full cell spreading and efficient cell migration (*18*). However, the relevance of engaging the entire talin-rod domain and the role of this association for the maintenance of established FAs remained unclear. Potential, alternative molecular linkages were identified—such as kindlin (*31*), α-actinin (*32*), or tensin (*33*)—that could act as redundant connectors between integrins and the actin cytoskeleton, once integrin receptors are activated and the overall adhesion structure has formed. To address this issue, we generated two talin constructs: Tln-AsLOV2, in which mCherry-AsLOV2 is C-terminally fused to the talin-1 head domain (amino acids 1 to 447) (Fig. 2A), and Zdk2-Tln, in which Zdk2-YPet is linked to the N-terminal end of the talin-1 rod domain (amino acids 448 to 2541) (Fig. 2B). Both constructs were stably expressed in Tln1^−/−^Tln2^−/−^ fibroblasts genetically depleted of talin-1 and talin-2 (TlnKO cells), which are characterized by a severe cell adhesion and spreading phenotype. As a control construct, we used talin-1 with YPet inserted after amino acid 447 (Tln-YPet) (Fig. 2C) that restored the phenotype of TlnKO cells (Fig. 2, D and E), as shown before (*6*, *7*). Expression of either Tln-AsLOV2 or Zdk2-Tln alone failed to induce cell spreading and the formation of FAs (Fig. 2, F and G). By contrast, cells expressing both parts of talin-1 forming an OM linkage (Tln-447-OM) showed FA formation and cell spreading (Fig. 2, H and I, and fig. S2A). Vinculin, which is thought to require preceding talin tension for efficient recruitment (*34*, *35*), localized to FAs of Tln-YPet– and Tln-447-OM–expressing cells. Furthermore, the phosphorylation of FA kinase (FAK) at tyrosine-397, a hallmark of mechanochemical signalling at FAs (*36*), was evident in both cell lines (fig. S2B).

### Sudden disengagement of the talin-actin linkage reveals its crucial role for FA integrity

To test whether the engineered talin linkages can be light-modulated, we analyzed Tln-447-OM–expressing cells by live-cell imaging. Consistent with the absorption spectrum of AsLOV2 (*37*), we could excite YPet (at 515 nm) and mCherry (at 561 nm) to visualize both talin fragments in FAs without disrupting the AsLOV2-Zdk2 interaction and inducing morphological changes in cells. However, exposing cells to short light pulses at 458 nm, with each light pulse corresponding to a light intensity of about 3.2 mW/cm^2^, frequently induced a rapid disintegration of FAs and collapse of cells, indicated by a sudden reduction in cell size (Fig. 2, J and K, and movie S1). The sudden collapse was often accompanied by a spatial separation of YPet and mCherry signals at the light-stimulated adhesion sites, indicating that the actin-binding rod domain of talin-1 was pulled into the cell body, while the integrin-binding head domain remained at the initial site of adhesion. Co-staining with a plasma membrane marker (CellMask) showed that in many such cases, the talin-head domain was literally ripped out of the cell (fig. S3, A and B). To confirm the specificity of this effect, we generated a control construct using an AsLOV2(C450A, L514K, G528A, L531E, and N538E) mutant, which is insensitive to light modulation (Tln-AsLOV2I) (*26*). As expected, cells expressing the Tln-AsLOV2I–based dimer were unaffected by the light stimulation and did not change cell size over the course of the experiment (fig. S4, A and B, and movie S2).

**Fig. 3.**
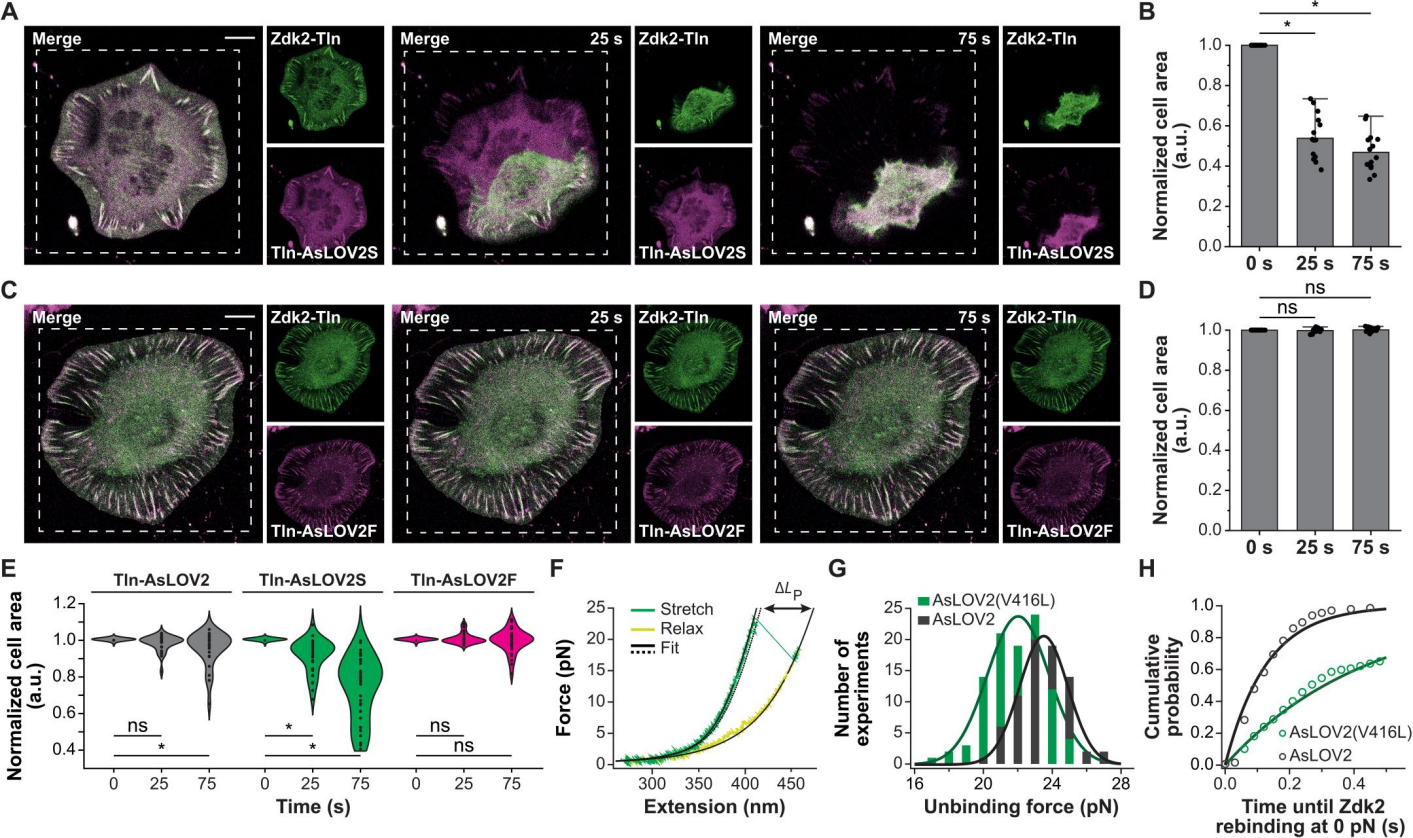
Controlling the talin junction using AsLOV2 variants. (**A**) Representative image series of a TlnKO cell expressing the slow-rebinding variant AsLOV2(V416L) (Tln-AsLOV2S) and Zdk2-Tln before and under light stimulation. The irradiation area is indicated by the white, dashed line. (**B**) Quantifying the normalized cell area shows that cells rapidly collapse upon stimulation (*n* = 15; paired sample Wilcoxon signed-rank test). (**C**) Image series of a TlnKO cell expressing the fast-rebinding variant Tln-AsLOV2(I427T) (Tln-AsLOV2F) and Zdk2-Tln before and under light stimulation. (**D**) Quantification of the normalized cell area suggests efficient rebinding after light stimulation preventing cell collapse and disintegration of FAs (*n* = 15; paired sample Wilcoxon signed-rank test). (**E**) Comparison of the normalized cell area of TlnKO cells expressing Tln-AsLOV2 (gray), Tln-AsLOV2S (green), and Tln-AsLOV2F (magenta) together with Zdk2-Tln (*n* = 31, 35, and 35; paired sample Wilcoxon signed-rank test). (**F**) Representative force-extension trace of the AsLOV2(V416L)-Zdk2 dimer. (**G**) Histogram of unbinding forces of AsLOV2 (black) and AsLOV2(V416L) (green) from Zdk2; slightly lower unbinding forces are observed for AsLOV2(V416L) (22 pN; *n* = 100) as compared to AsLOV2 (24 pN; *n* = 75). The histogram was fit by a Gaussian. (**H**) Cumulative probability versus time of Zdk2 rebinding at 0 pN for AsLOV2-Zdk2 (black) and AsLOV2(V416L)-Zdk2 (green) pairs; data were fit by a single exponential function. AsLOV2 rebinds faster (0.13 s; *n* = 134) than the AsLOV2(V416L) mutant (0.44 s; *n* = 471). Scale bars, 10 μm. *P < 0.05; ns, *P* ≥ 0.05.

Intriguingly, we did not observe a collapse in all light-stimulated cells expressing Tln-447-OM. A plausible explanation was that rapid rebinding of AsLOV2-Zdk2 in noncollapsing cells was sufficient to maintain cell adhesion. To test this hypothesis, we generated two AsLOV2 mutants previously shown to affect the AsLOV2-Zdk association kinetics (*26*). Cells reconstituted with a construct based on AsLOV2(V416L), which is expected to slow down dimer rebinding (Tln-AsLOV2S), showed a substantial increase in collapse frequency after light stimulation (Fig. 3, A and B, and movie S3). By contrast, cells reconstituted with the fast-rebinding AsLOV2(I427T) mutant (Tln-AsLOV2F) did not collapse upon irradiation, presumably because linkages were quickly reconstituted between light pulses (Fig. 3, C to E, and movie S4). To test whether the increased sensitivity of the Tln-AsLOV2S–expressing cells was indeed the result of slower rebinding, we expressed and purified the respective calibration construct for single-molecule force spectroscopy analysis. This mutant showed a Zdk2 unbinding pattern similar to that of the wild-type construct, with averaged unbinding forces at about 22 pN (Fig. 3, F and G, and fig. S1B). Consistent with the slight reduction in unbinding force, the average lifetime of the peptide-bound state under force was shortened for AsLOV2(V416L) when compared to AsLOV2 (0.18 s versus 0.56 s; fig. S1C), while rebinding at zero force was significantly delayed (0.44 s as compared to 0.13 s; Fig. 3H). Thus, optomechanically engineered linkages can be disengaged with light, and the degree of rebinding can be tuned using different AsLOV2 variants.

### Molecular optomechanics allows spatiotemporally controlled and reversible modulation of mechanical linkages

**Fig. 4.**
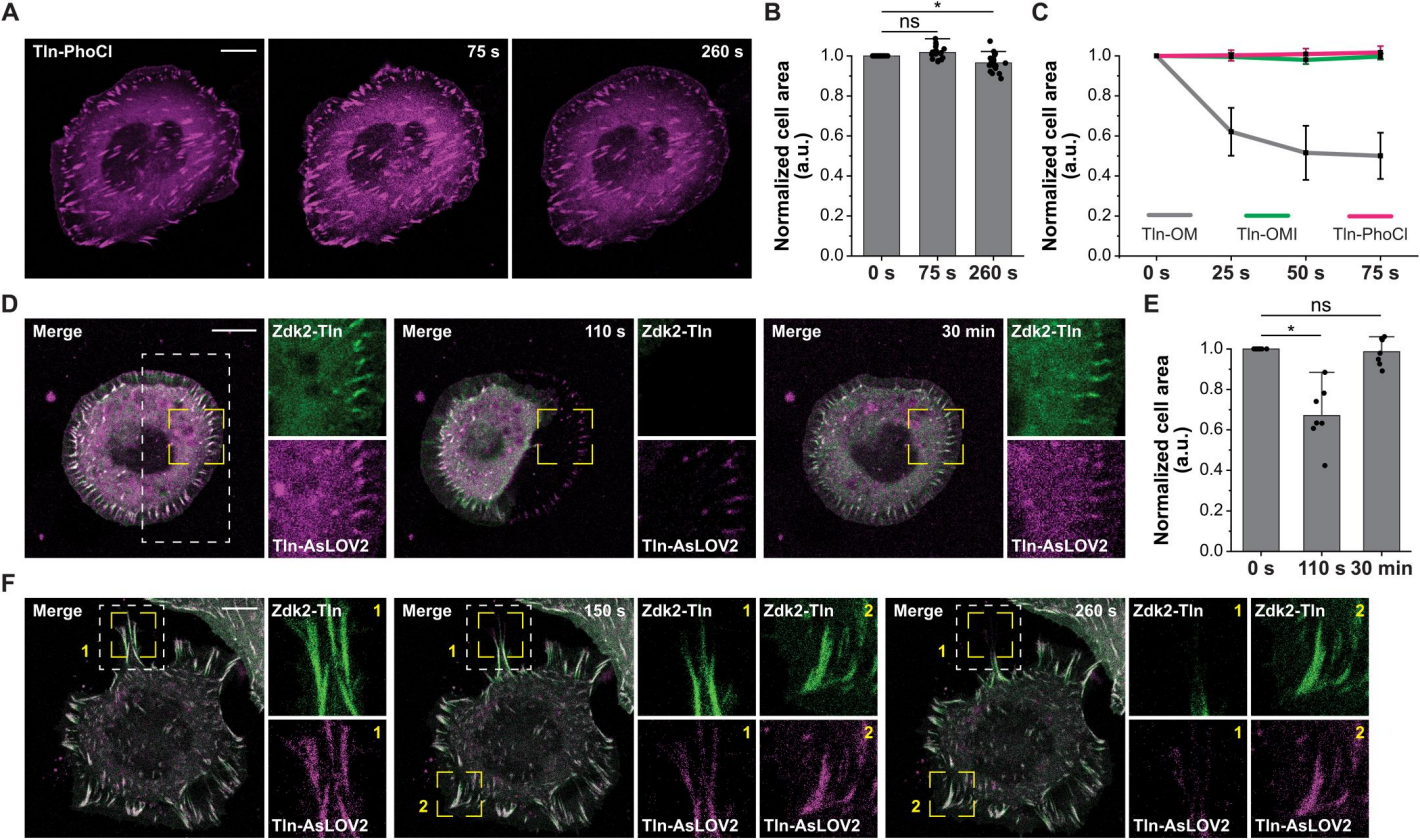
Talin optomechanics requires low light doses, allows reversible measurements, and provides high spatiotemporal control. (**A**) Representative image of a living TlnKO cell expressing Tln-PhoCl before and under light stimulation with 405 nm. (**B**) Quantification reveals a slight reduction in cell area after prolonged (260 s) exposure to 405-nm light (*n* = 18; paired sample Wilcoxon signed-rank test). (**C**) Comparison of the normalized cell area medians of cells expressing the Tln-447-OM construct (Tln-OM; gray), the light-insensitive control with Tln-AsLOV2I (Tln-OMI; green), or Tln-PhoCl (magenta). Note that Tln-PhoCl was stimulated with 405-nm light, while Tln-447-OM and Tln-OMI were stimulated with 458 nm. (**D**) Representative image of a living TlnKO cell expressing Tln-AsLOV2 (magenta) and Zdk2-Tln (green) before and after light stimulation. Note that irradiation indicated by the white dashed line was only used initially to induce cell collapse. Zoomed-in areas are indicated by yellow dashed lines. (**E**) Quantification demonstrates full recovery of cell area within 30 min (*n* = 7; paired sample Wilcoxon signed-rank test). (**F**) Image of a TlnKO cell expressing Tln-AsLOV2 and Zdk2-Tln before and after the start of light stimulation at individual FAs. The irradiation area is indicated by the white, dashed line; zoomed-in areas 1 (stimulated) and 2 (nonstimulated) are indicated by yellow dashed lines. Note that only light-stimulated FAs retract (*1*), whereas unstimulated FAs (*2*) remain unaffected. Scale bars, 10 μm. *P < 0.05; ns, *P* ≥ 0.05.

Next, we examined how the technique compares to alternative, optogenetic approaches. The only existing, genetically encoded method enabling the controlled disengagement of molecular linkages in cells is based on a photocleavable protein named PhoCl (*20*). Although PhoCl and the approach described here are based on fundamentally different principles (i.e., photocleavage versus light-induced unbinding) and are likely to be used in different experimental contexts, we wanted to benchmark our method against the current state-of-the-art and integrated PhoCl-mCherry into talin-1 using the same insertion site after amino acid 447 (Tln-PhoCl). Expression of this construct efficiently restored the adhesion and spreading defect of TlnKO cells confirming that PhoCl is capable of withstanding the mechanical forces associated with cell adhesion formation (Fig. 4A). As PhoCl responds to UV light, we stimulated cells with 405 nm but failed to detect a morphological response or a collapse of individual FAs, even after the illumination time was extended to 4 min (Fig. 4, A to C, and movie S5). We also tested a recently developed and presumably more sensitive variant (*21*), PhoCl2c (Tln-PhoCl2c), and observed instances where isolated FAs visibly retracted after prolonged 405-nm stimulation (fig. S5, A and B, and movie S6). However, cells expressing Tln-PhoCl2c never collapsed, and similar retraction effects were also observed in control cells expressing Tln-YPet (fig. S5, C and D). This suggests that responses in PhoCl2c were nonspecific in our experiments and probably caused by the high UV light intensities, which were approximately 300 times stronger (about 1000 mW/cm^2^) than those used in OM-based experiments.

A further difference between the approach described here and the PhoCl-based technique, as well as approaches using photocleavable chemical compounds, is the reversibility of the modulation, allowing the reconstitution of the broken molecular linkages. To demonstrate that cells expressing Tln-447-OM are able to recover from the light-stimulated collapse, we performed live-cell imaging focussing on isometrically spread cells and disrupted talin linkages in only half of the cell area. This approach typically induced the collapse of the light-treated part, again characterized by the separation of YPet and mCherry signals, whereas the untreated half of the cell was morphologically unaffected (Fig. 4, D and E, and movie S7). Monitoring both channels over time revealed the formation of new adhesion sites with colocalizing YPet and mCherry signal within tens of seconds after stopping light stimulation; FA reformation seemed especially efficient in areas where cell adhesion had been established before. By the end of the experiments, typically at around 30 min after the light-induced collapse, cells had regained their initial shape. Last, we tested whether we could modulate talin-1 linkages in individual FAs and focussed on cells with clearly separable cell adhesion structures. Disengaging talin-1 linkages in these isolated FAs often led to a specific collapse of the selected cell adhesion complex within seconds after starting light stimulation, while nonirradiated FAs in the same cell were unaffected (Fig. 4F and movie S8).

Together, these results demonstrate that the AsLOV2-Zdk2–based molecular optomechanics approach allows a reversible modulation of force-bearing, molecular linkages in living cells with high spatiotemporal control using comparably low light intensities. Our experiments reveal that the mechanical engagement of talin-1 through its talin-rod domain is indispensable for the maintenance of cell-matrix adhesion complexes and the integrity of the overall cell morphology. Under the conditions tested here, the mechanical function of talin is unique and cannot be compensated by other force-bearing molecules.

### Loss of talin engagement through actin-binding site 3 exclusively affects peripheral FAs

**Fig. 5.**
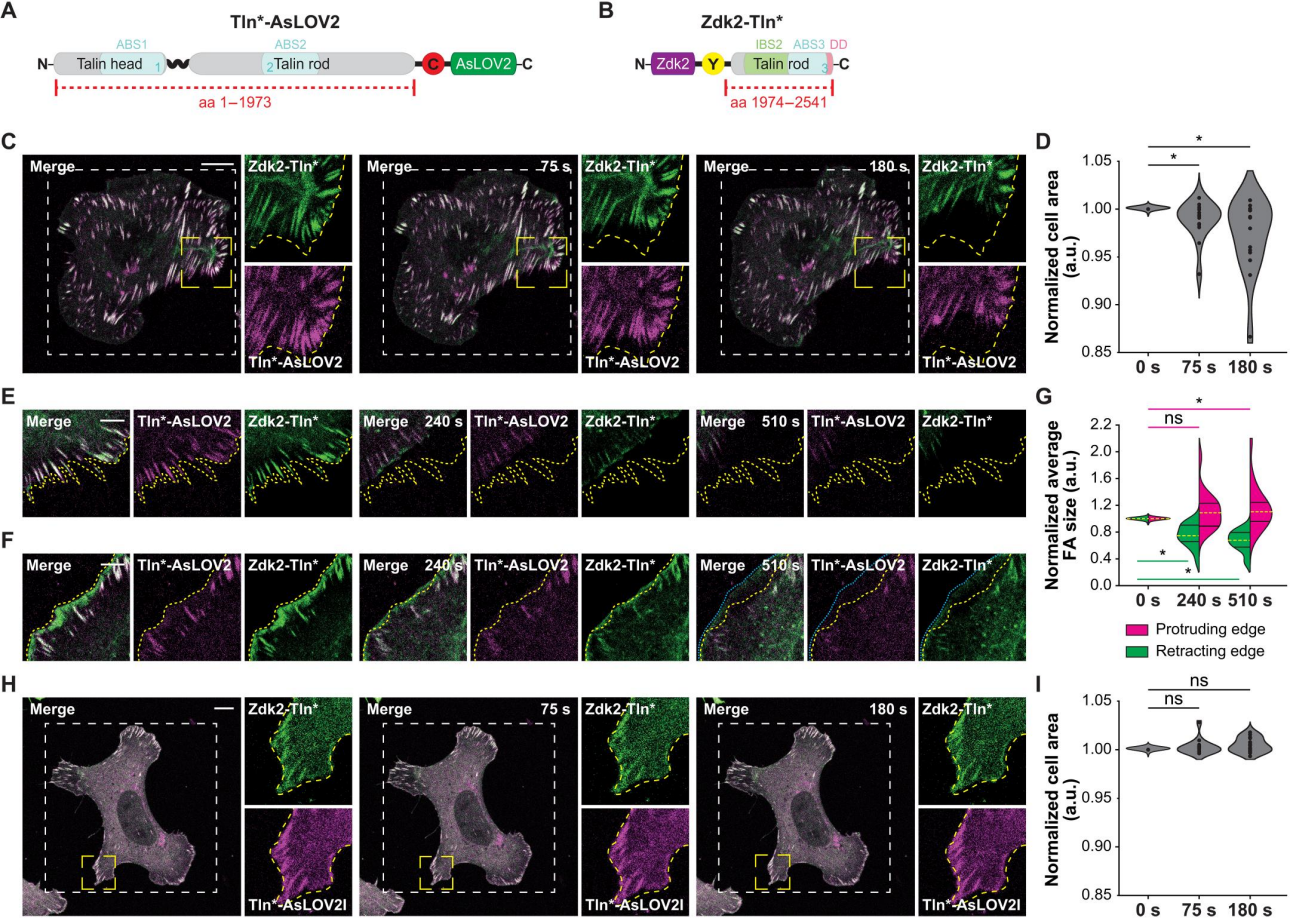
Molecular optomechanics indicates mechanical role of talin’s C-terminal motifs. (**A** and **B**) Scheme of expression constructs showing the N-terminal talin-1 fragment (amino acids 1 to 1973) tagged with mCherry-AsLOV2 (Tln*-AsLOV2) (A) and the C-terminal talin-1 fragment (amino acids 1974 to 2541) fused to Zdk2-YPet (Zdk2-Tln*) (B). Locations of ABS1, ABS2, ABS3, IBS2, and the talin dimerization domain are indicated. (**C**) Image series of a living TlnKO cell expressing Tln*-AsLOV2 (magenta) and Zdk2-Tln* (green) before and under light stimulation. Irradiation area is indicated by the white dashed line, zoomed-in area is outlined by yellow lines, initial cell edge position is indicated by yellow dashed line. (**D**) Quantification of the normalized cell area reveals a small but significant reduction under light stimulation (*n* = 15; paired sample Wilcoxon signed-rank test). (**E** and **F**) Image series of a retracting (E) and a protruding (F) edge of a Tln-1973-OM cell under prolonged light stimulation. Note changes in cell edge position between the initial (yellow lines) and the final time point (blue lines) (F). (**G**) Quantification of the normalized average FA size in retracting (green) and protruding (magenta) edges. (**H**) Image series of a living TlnKO cell expressing the light-insensitive Tln*-AsLOV2I (magenta) and Zdk2-Tln* (green) constructs before and under light stimulation. (**I**) Quantification of the normalized cell area in control cells before and under light stimulation (*n* = 14; paired sample Wilcoxon signed-rank test). Scale bars, 10 μm (C and H) and 5 μm (E and F) *P < 0.05; ns, *P* ≥ 0.05.

The results so far prompted us to explore the talin-actin linkage in more detail. The talin-rod domain contains multiple interaction sites to connect with the actin cytoskeleton. In addition to numerous vinculin binding motifs, it harbors two actin-binding sites (ABS2 and ABS3), a second integrin-binding site (IBS2), and a talin dimerization motif at its C-terminal end (*22*). How these motifs contribute to the mechanical connection is still unclear. Therefore, we decided to disengage ABS3, IBS2, and the dimerization motif of talin but allow for talin-actin engagement through ABS2. For this, we engineered talin linkages in which the OM module was integrated at a previously identified insertion site within the talin-rod domain (*7*), after amino acid 1973, using two constructs: Talin-1(1 to 1973)–mCherry–AsLOV2 (Tln*-AsLOV2) and Zdk2–YPet–Talin-1(1974 to 2541) (Zdk2-Tln*) (Fig. 5, A and B). Consistent with the previous experiments, TlnKO cells expressing both fragments forming Tln-1973-OM adhered to FN-coated surfaces, displayed FAs, and underwent cell spreading. However, they did not collapse when exposed to the same 458-nm light doses, which were used in the experiments with Tln-477-OM. The mCherry/YPet signals never abruptly separated, and cells maintained their overall morphology, while large peripheral FAs often retracted over the course of minutes (Fig. 5, C and D, and movie S9). Extending 458-nm light exposure to 510 s still did not induce a sudden collapse of cells, and new adhesions were able to form and support the extension of the plasma membrane in protrusive areas of the cells (Fig. 5, E to G, and fig. S6). The retraction of FAs was not observed in cells expressing the corresponding light-insensitive Tln*-AsLOV2I control (Fig. 5, H and I, and movie S10).

To specify whether the retracting adhesion were FAs or so-called fibrillar adhesions, which emerge from FAs but are less sensitive to mechanical forces (*38*), we performed immunostainings using antibodies against β_1_ integrin and phospho-tyrosine (p-tyrosine). Fibrillar adhesions are distinguished from FAs by low p-tyrosine levels, yet all large adhesions in our cells displayed robust p-tyrosine signal. This indicates that the large adhesion structures retracting after irradiation are FAs and not fibrillar adhesions (fig. S6).

Together, the results suggest that the C-terminal ABS3, IBS2, and the dimerization motif of talin-1 are not essential to maintain the overall integrity of the cell and the formation of new FAs in lamellipodia-like protrusions. Instead, these C-terminal motifs seem to play a specific role for stabilizing large FAs at the cell periphery.

### Abrupt loss of the desmoplakin linkage leads to the disruption of cell-cell adhesion under stress

**Fig. 6.**
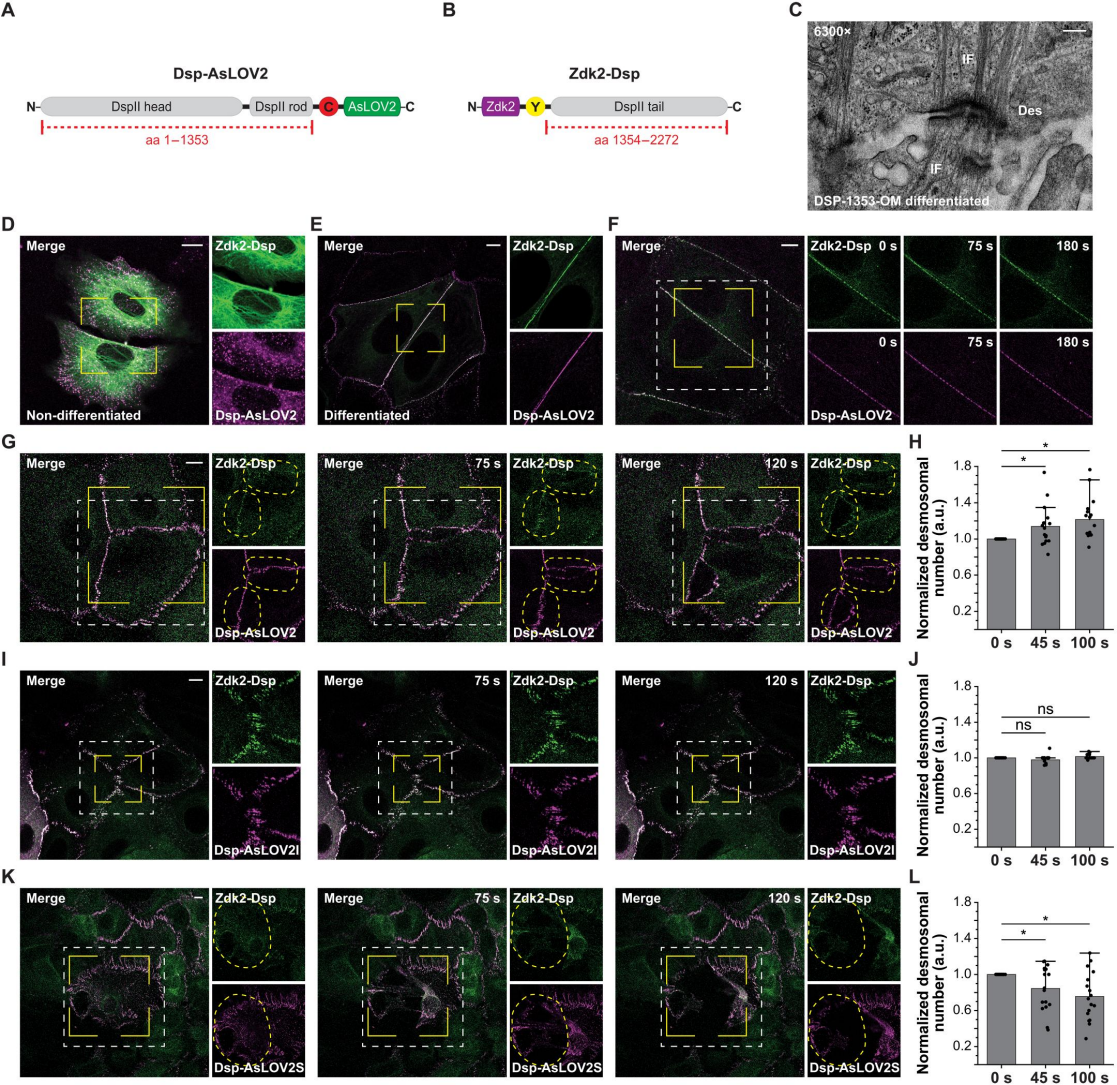
Applying molecular optomechanics to Dsp. (**A** and **B**) Scheme of DspII expression constructs showing DspII (amino acids 1 to 1353) tagged with mCherry-AsLOV2 (Dsp-AsLOV2) (A) and the DspII (amino acids 1354 to 2272) fused to Zdk2-YPet (Zdk2-Dsp) (B). (**C**) EM image of a differentiated Dsp-1353-OM cell showing prominent association of desmosomes (Des) with IFs. (**D** and **E**) Representative images of living DspKO MEKs expressing Dsp-AsLOV2 (magenta) and Zdk2-Dsp (green) before (D) and after (E) differentiation. Zoomed-in areas are outlined by yellow lines. (**F**) Image series of a DspKO MEK expressing Dsp-AsLOV2 (magenta) and Zdk2-Dsp (green) before and under light stimulation. The area of irradiation is indicated by the white dashed line. (**G**) Image series of a DspKO MEK expressing Dsp-AsLOV2 (magenta) and Zdk2-Dsp (green) light-stimulated and simultaneously subjected to mechanical pulling. Note the separation of junctional signals (yellow dashed line). (**H**) The number of desmosomal clusters increase as the signal of intercellular junctions is split into two (*n* = 17; paired sample Wilcoxon signed-rank test). (**I**) Image series of a DspKO MEK expressing the light-insensitive control Dsp-AsLOV2I (magenta) and Zdk2-Dsp (green). (**J**) Quantifying desmosomal clusters reveals no change in controls (*n* = 15; paired sample Wilcoxon signed-rank test). (**K**) Image series of a DspKO MEK expressing the slow-rebinding variant Dsp-AsLOV2S (magenta) with Zdk2-Dsp (green). Light-stimulation and simultaneous pulling leads to a rapid breaking of intercellular junctions. (**L**) Quantification confirms disintegration of cell-cell junctions upon light stimulation and pulling (*n* = 17; paired sample Wilcoxon signed-rank test). Scale bars, 0.5 μm (C) and 10 μm (D to K). *P < 0.05; ns, *P* ≥ 0.05.

We wanted to test whether the molecular optomechanics approach can be applied to proteins in other cellular structures and targeted a major component of desmosomes called desmoplakin (Dsp). Desmosomes are cadherin-based complexes essential for maintaining the coherence of neighboring cells and thus the physical integrity of many tissues, in particular, the epidermis and the heart muscle (*25*, *39*). Dsp mediates the engagement between desmosome-specific cadherins and the intermediate filament (IF) system, but it is still not fully understood under which conditions it becomes relevant as a mechanical linker. Thus, we generated expression constructs for two DspII fragments—DspII(1 to 1353)-mCherry-AsLOV2 (Dsp-AsLOV2) and Zdk2-YPet-DspII(1354 to 2272) (Zdk2-Dsp) (Fig. 6, A and B)—using an insertion site that would allow for the separation of desmosomes from IFs without affecting the function of the Dsp molecule (*12*). We expressed both constructs in Dsp-deficient (DspKO) murine epidermal keratinocytes (MEKs), differentiated them through addition of Ca^2+^, and used electron microscopy (EM) analysis to confirm that desmosomes were efficiently connected to IFs (Fig. 6C). Live-cell imaging of nondifferentiated cells revealed that both Dsp fragments were rather diffusely distributed in the absence of desmosomes and seemed to associate with the keratin network (Fig. 6D), while both DspII fragments efficiently colocalized at cell-cell junctions upon differentiation (Fig. 6E). Intercellular junctions remained intact, and no obvious separation between cells was apparent when cells were stimulated with 458 nm, using similar light intensities as before (Fig. 6F and movie S11).

Next, we exerted mechanical forces on the desmosomes by pulling cells with a microneedle at a velocity of 1 μm/s, a speed that is typically associated with naturally occurring molecular processes in cells, such as the movement of motor proteins (*40*). Under these conditions, we observed rapid breaking of intercellular junctions upon light stimulation (Fig. 6, G and H, and movie S12). These disruptions did not occur in cells expressing the light-insensitive control construct Dsp-AsLOV2I (Fig. 6, I and J, and movie S13). As expected, the degree of cell-cell contact disruption under stress increased when the Dsp junction was reconstituted with the dimer containing the slow-rebinding AsLOV2(V416L) mutant (Dsp-AsLOV2S). In these conditions, external stress and simultaneous light stimulation often led to a complete loss of cohesion between cells. As a result, Dsp signals were often retracted together with rapidly collapsing cells leading to a reduction in the number of detected desmosomes (Fig. 6, K and L, and movie S14).

These experiments reveal that the Dsp-mediated connection between desmosomes and the keratin network is dispensable for the stability of epithelial cell-cell adhesions under homeostatic conditions, presumably because other adhesion proteins in adherens junctions or tight junctions can compensate and bear mechanical loads. However, application of external forces and simultaneous fracture of the Dsp linkage leads to disruption of intercellular junctions revealing its important role as a mechanical linker under stress conditions.

## DISCUSSION

The ability of subcellular complexes, such as cell-matrix or cell-cell adhesion, to transduce mechanical signals is critical for development and tissue homeostasis. An important step toward understanding the underlying molecular processes was the establishment of force-sensing techniques to identify proteins, which are directly exposed to mechanical signals (*4*, *8*, *41*). However, the interpretation of these experiments was challenging because most subcellular structures comprise many different molecules—in the case of cell adhesions tens to hundreds (*42*, *43*)—that could potentially compensate for each other rendering individual molecular bonds redundant. In this study, we addressed this issue by establishing a new molecular optomechanics approach.

Key to the methodology is the mechanical stability of the optogenetic dimer, AsLOV2-Zdk2, that withstands mechanical forces of up to 24 pN and remains bound for minutes, even when exposed to constant mechanical loads. Note that the module is significantly more stable than previously introduced, conceptually similar constructs (*18*, *44*). For instance, an FKBP-FRB dimer, which can be controlled by the addition of rapamycin, withstands forces of around 5 pN (*44*), while a light-controlled LOV2-SsrA-SspB module resists forces of up to 10 pN for about 1 to 10 s (*18*). However, individual molecules in cells can be exposed to forces higher than 10 pN—as shown for integrins, the Notch receptor, metavinculin, talin-1 and talin-2—and are likely required to withstand such loads for tens of seconds (*6*, *9*, *27*). Thus, introducing an OM module able to resist these mechanical stresses should substantially expand the range of potential experiments, which is exemplified in our first application to talin. Previous studies already pointed toward a critical role for mechanically engaging the talin-rod domain, yet focused on controlling the C-terminal region of the protein (dimer insertion after talin-rod domain R10), where mechanical forces are comparably low (*7*, *18*, *44*). The AsLOV2-Zdk2–based approach developed here enabled us to control the mechanical engagement of the entire talin-rod domain (dimer insertion before R1), which is exposed to higher mechanical tension (*7*).

It was already established that talin is essential for integrin activation mediated by its head domain (*23*, *28*), and we had demonstrated that the talin rod domain forms a force-bearing linkage to the actin cytoskeleton (*6*, *7*). The previously mentioned OM study showed that engaging the C-terminal part of the talin-rod domain with light promotes the formation of new FAs, cell polarization, and migration (*18*). However, it remained unclear to which degree the ongoing cytoskeletal engagement of talin is required for maintaining already established cell adhesion, since FAs contain numerous proteins that could form alternate connections between integrin receptors and actin networks (e.g., α-actinin, kindlin, tensin, and filamin). Our data show that none of these linker molecules compensate for a sudden loss of the talin connection, at least under the conditions tested here. Disengaging the entire talin-rod domain leads to a remarkably fast disintegration of FAs, often resulting in the collapse of cells within tens of seconds. This demonstrates that the mechanical role of talin is not only restricted to initial phases of cell adhesion formation—through integrin activation and establishing a first mechanical connection to the cytoskeleton—but also crucial for the long-term maintenance of cell-matrix adhesions.

By contrast, disengaging specifically the C-terminal ABS3, IBS2, and the dimerization motif of talin has comparably mild consequences for cell adhesion integrity and appears to affect exclusively the stability of large, peripheral FAs. These results seem consistent with the observation that mechanical tension at the C-terminal end of talin is low (*7*) and that mutation of talin’s ABS3 reduces mechanical loads specifically in peripheral adhesion structures (*29*). It is therefore tempting to speculate that these domains have a more specialized function during cell adhesion, for example, to coordinate rear retraction during mesenchymal cell migration.

The OM experiments on Dsp reveal a very different role and further underline the molecular complexity governing cell-matrix and cell-cell adhesion mechanics. Instant loss of the Dsp-based linkages does not impair the mechanical integrity of cell-cell junctions, as long as cells are not exposed to external mechanical forces. By contrast, application of comparably mild external stress and simultaneous, light-induced breaking of the Dsp linkage leads to a severe disruption of cell-cell adhesion. While our experiments lack the resolution to unequivocally determine where the disruption in the desmosome occurs, our movies seem to suggest that entire desmosomes can be pulled into one of the neighboring cells, an observation reminiscent of a recent study showing the internalization of intact desmosomes during cell scattering (*45*). Overall, these results seem particularly relevant in light of clinical observations where altered Dsp function impairs tissue integrity, for example, causing epidermolysis bullosa in the skin or leading to cardiomyopathy in heart muscle tissue (*46*–*48*). Our data support the notion that a major cause for these debilitating disorders is the loss of Dsp’s function as a mechanical linker. Since a related and clinically highly relevant condition called pemphigus is also caused by a loss of established desmosomes, it will be exciting to apply AsLOV2-Zdk2–based optomechanics to desmogleins, which are targeted by autoantibodies that promote disease progression (*49*).

In summary, the method described here constitutes a new approach to investigating mechanobiological processes with molecular specificity and high spatiotemporal control in living cells. Its ability to withstand substantial mechanical loads under nonstimulating conditions, its inherent reversibility, and sensitivity for low light doses outside the UV spectrum should make this genetically encoded technique an efficient tool to clarify the role of many force-bearing proteins in cells.

## MATERIALS AND METHODS

### Antibodies and reagents

The following antibodies and reagents were used for cell culture experiments: anti-vinculin (Sigma-Aldrich, V9131; IF; 1:200), anti-FAK (pY397) (Thermo Fisher Scientific, 44-624G; IF; 1:400), anti–integrin β_1_ (9EG7) (BD Pharmingen, 550531; IF; 1:200), anti–p-tyrosine (4G10) (Millipore, 05-1050X; IF; 1:200), anti-mouse immunoglobulin G (IgG) Alexa Fluor 405 (Thermo Fisher Scientific, A31553; IF; 1:200), anti-mouse IgG Alexa Fluor 647 (Thermo Fisher Scientific, A21235; IF; 1:500), anti-rabbit IgG Alexa Fluor 647 (Thermo Fisher Scientific, A21244; IF; 1:500), anti-rat IgG Alexa Fluor 647 (Thermo Fisher Scientific, A21247; IF; 1:500), CellMask Deep Red plasma membrane stain (Thermo Fisher Scientific, C10046; Live stain; 1:1000), collagen I (Corning, 354236), fibronectin (Merck, 341631), puromycin (Sigma-Aldrich, P8833), and hygromycin B (Sigma-Aldrich, H3274).

### Generation of expression constructs

Our previously published protocols (*50*), using NEBuilder HiFi DNA Assembly Master Mix (New England Biolabs, E2621L), were used to generate all expression constructs in this study. The following cDNA templates were used: human talin-1 (GeneBank BC042923), mCherry-AsLOV2 (Addgene #81041), mCherry-AsLOV2(I427T) (Addgene #81035), Zdk2 (Addgene #81011), YPet (pCEP4-YPet; amino acids 1 to 228), PhoCl-mCherry (Addgene #87691), PhoCl2c-mCherry (Addgene #164037), and human DspII (Addgene #32227). Final cDNA sequences were assembled in expression vectors pLPCX or pLHCX (Clontech, 631511), N-terminal fragments of the dimers and full-length constructs in the former, and C-terminal fragments in the latter. Mutations in AsLOV2(V416L) and AsLOV(C450A, L514K, G528A, L531E, and N538E) were also created using the Gibson assembly approach. The correct cDNA sequences were confirmed by DNA sequencing (Microsynth Seqlab).

### Generation, expression, and purification of single-molecule constructs

For single-molecule force spectroscopy–based calibration, mCherry-AsLOV2 and Zdk2-YPet were connected by a 126–amino acid–long glycine-glycine-serine (GGS)_42_ linker to allow for repeated measurements of binding/unbinding events. Short ybbR tags were added before mCherry and after YPet sequences to provide handles for DNA attachment. A histidine (His_6_)-tag was included at the C-terminal end to enable the purification of the fusion protein. The resulting [ybbR-mCherry-AsLov2-GGS_42_-Zdk2-YPet-ybbR-His_6_] construct was assembled in the pLPCX expression vector and transfected into human embryonic kidney (HEK) 293 cells using a CaPO_4_ precipitation protocol, as described earlier (*51*). After 48 hours, cells were harvested, resuspended in hypotonic lysis buffer [20 mM tris and 2 mM MgCl_2_ (pH 7.4)], incubated for 20 min on ice, and then lysed with a Dounce homogenizer. Cell lysates were cleared by 10-min centrifugation at 4°C and purified using metal ion affinity chromatography (His-Trap; GE Healthcare). Purified samples were concentrated to about 20 μM by membrane ultrafiltration (Vivaspin; GE Healthcare) and stored at −80°C in phosphate-buffered saline (PBS; pH 6.7).

### Assembling protein-DNA conjugates

To generate a protein-DNA construct for optical tweezers experiments, single-stranded (ss) oligonucleotides (oligos) functionalized with Coenzyme A (CoA) (5′-GGCAGGGCTGACGTTCAACCAGACCAGCGAGTCG-3′-CoA; Biomers) were bound covalently to the first serine of the terminal ybbR tags by a specific catalytic reaction using the Sfp enzyme. The oligo attachment was performed with 5 nmol of protein and 10 nmol of CoA-oligos in Sfp buffer [50 mM Hepes, 10 mM MgCl_2_, 0.5 μM tris(2-carboxyethyl)phosphine (TCEP), and 7 μM Sfp enzyme (pH 7.5)] for 2 hours on ice. The sample was subsequently purified by size exclusion chromatography (Yarra SEC 3000, 3 μm, 300 × 7.8 mm; Phenomenex) in PBS + 5 μM TCEP (pH 7.4). Protein with oligo strands attached on both ends was concentrated to ssDNA (about 22 ng/μl) by membrane ultrafiltration (Amicon Ultra; Sigma-Aldrich/Merck) and stored at −80°C. Then, 16 ng of oligo-protein complex was hybridized with about 250 ng of in-house synthesized double-stranded DNA handles for 1 hour on ice, resulting in the final DNA-protein-DNA hybrid (POH). The DNA handles (template: bases 3643 to 4187 of the λ-phage genome) had an ss 5′-overhang complementary to the CoA-oligo sequence on one end and were functionalized with either three biotin or digoxigenin molecules on the other end.

### Single-molecule force spectroscopy and data analysis

The POH solution was diluted about 1:1000 before it was added to anti-digoxigenin–coated 1-μm-sized beads (in-house functionalized AD bead; Bangs Laboratories). Silica beads were used for experiments in static chambers, and polystyrene beads were favored for experiments in a microfluidics setup. Trap experiments were performed with a commercial dual-beam optical tweezer setup (Ctrap; Lumicks). The measurement buffer [PBS and 1 mM TCEP (pH 7.4)] was supplemented with an oxygen scavenger system [1× pyranose oxidase (Sigma-Aldrich) or glucose oxidase (Sigma-Aldrich), 1× catalase (Serva), and 0.66% glucose]. To assemble the dumbbell-like configuration, in which a single protein is tethered between two beads, one POH-bound AD bead was trapped in the focus of the fixed trap and one streptavidin-coated bead (in-house functionalized SA bead; Bangs Laboratories) was held in the focus of the mobile trap. By moving the mobile trap toward the fixed one, beads were brought into close proximity to form a single tether between them.

In constant velocity experiments, the mobile trap was moved back and forth, so that the construct was stretched (unfolding process) and relaxed (refolding process). Experiments were performed with a constant pulling velocity of 200 and 500 nm/s with a waiting time of 0.2 to 0.6 s between each cycle. Recorded data were fit by a combination of the Worm-like chain (WLC) model and the extensible WLC formula to describe the protein unfolding and stretching of DNA (*52*, *53*). The trap stiffness calculated from corrected power spectra was about 0.32 pN/nm. Constant distance experiments at 11 and 16 pN were performed under continuous application of force to the construct. Jump experiments were executed within 0.03 s (cycle up) and 0.03 s (cycle down) by jumping fast between 0 and 20.5 ± 1 pN to obtain unbinding and rebinding lifetimes of the AsLOV2-Zdk2 dimer. To investigate AsLOV2-Zdk2 binding under light stimulation, a preinstalled 488-nm laser of the Ctrap instrument was used with an intensity of 3 and 30%. Since unbinding lifetimes were highly similar at both intensities, data obtained from these experiments were pooled. To analyze data, Igor Pro 8 (WaveMetrics) and Python were used.

### Cell culture conditions

Tln1^−/−^Tln2^−/−^ cells (TlnKO) and HEK293 cells were cultured in high-glucose Dulbecco’s Modified Eagle Medium (DMEM)–GlutaMAX medium (Thermo Fisher Scientific, 31966047) supplemented with 10% fetal bovine serum (FBS; Thermo Fisher Scientific, 10270-106) and 1% penicillin/streptomycin (P/S; Thermo Fisher Scientific, 15140122). For live-cell imaging, DMEM without phenol red but containing glucose (4.5 mg/ml), 25 mM Hepes, and 2 mM glutamine (all from Thermo Fisher Scientific) supplemented with 10% FBS and 1% P/S was used. Stable cell lines were established using the Phoenix cell transfection system. Ecotropic, retroviral particles were produced according to established protocols (*51*), and target cells were infected in the presence of polybrene (5 μg/ml). After five infection cycles, cells were selected with puromycin or hygromycin depending on the used expression vector (pLPCX or pLHCX). DspKO MEKs (a gift from K. Green, Northwestern University) were maintained in FAD medium: DMEM/Ham’s F12 (3.5:1.1) (PAN Biotech) without l-glutamine or sodium pyruvate but supplemented with NaHCO_3_ (3.096 g/liter), glucose (3.8548 g/liter), 10% Ca^2+^-depleted FBS, 2 mM GlutaMAX, 1 mM sodium pyruvate, 0.18 mM adenine, hydrocortisone (0.5 μg/ml), insulin (5 μg/ml), epidermal growth factor (10 ng/ml), 100 pM cholera toxin, and 0.5% P/S. MEKs were seeded on collagen I (50 μg/ml)–coated plasticware for cell culture. To induce the formation of desmosomes, FAD medium was supplemented with 1.2 mM CaCl_2_ (FAD^+^ medium).

### Live-cell imaging, light stimulation, micromanipulation, and cell area measurement

For live-cell imaging of reconstituted TlnKO cells, approximately 3.0 × 10^4^ cells were seeded onto imaging dishes (ibidi, 81158) coated with FN (10 μg/ml). Cells were allowed to spread overnight (O/N), and medium was exchanged to supplemented DMEM without phenol red. Light stimulation and image acquisition were carried out on a LSM880 confocal laser scanning microscope (Zeiss) equipped with a 63× immersion objective (LD LCI Plan-Apochromat 63×/1.2 Imm Corr DIC M27). Fluorophores were visualized using 514-nm (YPet) and 561-nm (mCherry) excitation. For light modulation of the AsLOV2-Zdk2 linkage, a 458-nm laser excitation pulse leading to about 3.2 mW/cm^2^ at the sample was used, typically with four iterations. Regions of interest (ROIs) for excitation were selected using the “Bleaching” feature in the Zen Black imaging software (Zeiss). For the analysis of MEKs, about 7.5 × 10^4^ cells were seeded onto glass bottom dishes (ibidi, 80137) coated with collagen I (50 μg/ml). Cells were allowed to spread O/N, and medium was exchanged to FAD^+^ to induce the formation of desmosomes. Micromanipulation experiments (pipette pulling) used a micromanipulator device (Eppendorf, TransferMan 4r) equipped with a glass microcapillary (Eppendorf, VacuTip I; 35°; 15 μm) set at an angle of 35°. The capillary tip was gently lowered onto cell nuclei and moved with a speed of 1 μm/s orthogonally from the cell-cell junction. Cell areas were evaluated using manually drawn ROIs in Fiji; cell boundaries were identified using the fluorescent signals of mCherry-AsLOV2 and Zdk2-YPet.

### EM sample preparation and imaging

For transmission EM, 6.5 × 10^4^ Dsp-1353-OM cells were seeded on 12-mm Ø glass coverslips placed in a 24-well plate. Cells were allowed to spread and form a confluent monolayer, and medium was then exchanged to FAD^+^ to induce the formation of desmosomes. Differentiated cells on the coverslips were washed and prefixed using a 4% paraformaldehyde (PFA) and 4% glutaraldehyde (GA) in 0.1 M cacodylate buffer (pH 7.4; supplemented with 0.1 M CaCl_2_) and then fixed in a 2% PFA and 2% GA in 0.1 M cacodylate buffer. Cells were postfixed in 1% osmium tetroxide and 1.5% potassium ferrocyanide in 0.1 M cacodylate buffer before dehydrating in increasing amounts of ethanol. Dehydrated cells were infiltrated and embedded in Epon-812 resin before sectioning in 50- to 55-nm slices, placed on grids, and stained with 1% uranyl acetate. Samples were imaged with a Zeiss Libra 120 transmission electron microscope at 80 kV.

### Region of interest isolation

For determining the FA areas, fluorescence images were imported into Fiji. A custom written routine was used to sequentially enhance image contrast (0.05%; normalize), median filter (radius, 2 pixels), autolocal threshold (method, Phansalkar; radius, 15; rest, default), despeckle, remove outliers (radius, 2 pixels; threshold, 50; which outliers, Bright), morphologically open (iterations, 1; count, 1), and analyze particles (size, 0.25 infinity μm^2^; exclude on edges). The generated ROI boundaries were verified by overlaying with the original fluorescence images and measuring FA areas within the cells (fig. S7); measured areas were normalized to those in the first frame. A similar routine was used to determine the amount of desmosomal clusters in fluorescence images of cell-cell junctions with the following changes: remove outliers (radius, 0.5 pixels; threshold, 50; which outliers, Bright) and no morphological opening (fig. S8). The amount of desmosomal clusters per frame was normalized to those from the first frame.

### Statistical analysis

Error bars indicate the SD if not indicated otherwise. Bar plots display the median value and the SD as error bars. For statistical evaluation of quantitative data, a two-sided Mann-Whitney *U* test (α = 0.05) or a paired sample Wilcoxon signed-rank test (α = 0.05) were used as indicated. Statistical significances were given by the *P* value: *P < 0.05; ns (not significant), *P* ≥ 0.05.
