# Supplementary material for: A molecular optomechanics approach reveals functional relevance of force transduction across talin and desmoplakin
Source: Sci Adv. 2023 Jun 21;9(25):eadg3347. doi: 10.1126/sciadv.adg3347 (PMC10284548; doi:10.1126/sciadv.adg3347)
Supplement: Supplementary file 1 — Figs. S1 to S8 Legends for movies S1 to S14 Legend for data S1 [file sciadv.adg3347_sm.pdf]

Science Advances
AAAS

Supplementary Materials for

A molecular optomechanics approach reveals functional relevance of force transduction across talin and desmoplakin

Tanmay Sadhanasatish *et al.*

Corresponding author: Carsten Grashoff, grashoff@uni-muenster.de

Sci. Adv. **9**, eadg3347 (2023)
DOI: 10.1126/sciadv.adg3347

The PDF file includes:

Figs. S1 to S8
Legends for movies S1 to S14
Legend for data S1

Other Supplementary Material for this manuscript includes the following:

Movies S1 to S14
Data S1

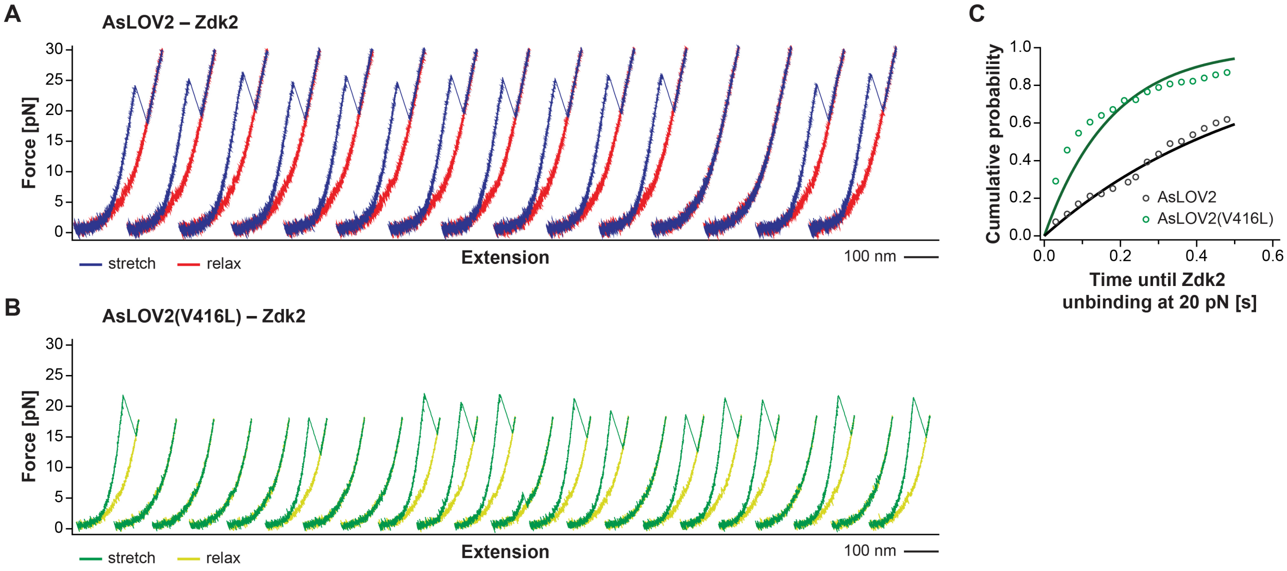

Fig. S1. Single-molecule force spectroscopy experiments of wildtype and mutant AsLOV2–Zdk2.

(**A**) Consecutive force-extension traces of one AsLOV2–Zdk2 dimer at a constant pulling velocity of 500 nm/s. Note that the Zdk2 peptide rebinds between almost every pulling cycle. Traces show a high reproducibility in the unbinding pattern and forces during stretching (blue) and relaxation (red). (**B**) Consecutive force-extension traces of one AsLOV2(V416L)–Zdk2 dimer at a constant pulling velocity of 500 nm/s. Note that the Zdk2 peptide frequently remains in the unbound state. Traces show a high reproducibility in the unbinding pattern, but unbinding forces can vary during the stretch (green) and relax (yellow) cycles. (**C**) Cumulative probability of bond rupture plotted against the time until Zdk2 unbinding at 20 pN. These data show that the mutant AsLOV2(V416L)–Zdk2 dimer unbinds faster than the wildtype at a force of 20 pN. Single exponential fits indicate bound lifetimes of 0.56 s for the wt (n=147) and 0.18 s for the V416L variant (n=478).

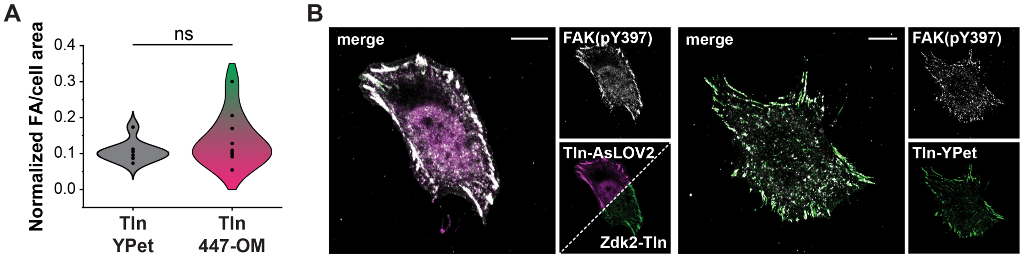

Fig. S2. Focal adhesion (FA) size analysis in reconstituted TlnKO cells.

(**A**) Quantification of FA area (normalized to cell area) of TlnKO cells expressing Tln-YPet or co-expressing Tln-AsLOV2 and Zdk2-Tln (Tln-447-OM) (n=9,10; Mann–Whitney U test). (**B**) Immunostaining of TlnKO cells expressing Tln-YPet or Tln-447-OM shows prominent FAK phosphorylation at Y397 in adhesions of both cell lines. Scale bars indicate 10 µm in all images. n.s. (not significant) $p \geq 0.05$.

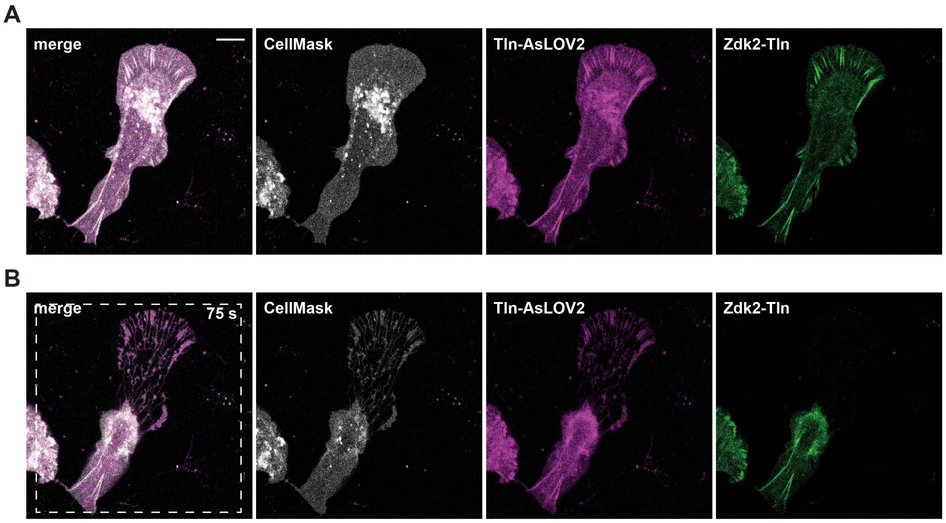

Fig. S3. Cellular collapse upon sudden abrogation of talin-1 linkages.

(**A** and **B**) Image series of a light-stimulated TlnKO cell co-expressing Tln-AsLOV2 (magenta) and Zdk2-Tln (green) before (**A**) and under (**B**) light-stimulation with 458 nm. The cell was additionally labelled with the CellMask dye to visualize the plasma membrane. The irradiation area is indicated by the white dashed line. Note in (**B**) that some of the Tln-AsLOV2 fragment is removed from the cell during collapse and remains – together with plasma membrane remnants – on the substrate, while the actin-associated Zdk2-Tln fragment (green) is pulled towards the cell interior. Scale bar indicates 10 µm.

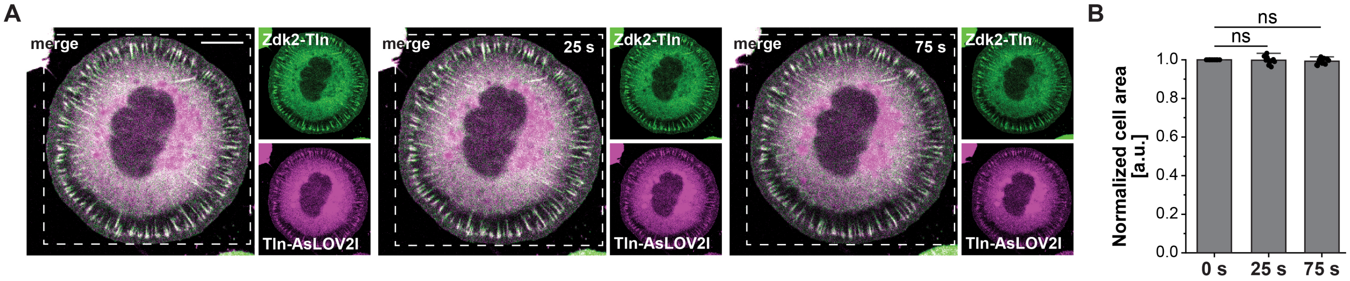

Fig. S4. Light-insensitive control cells do not display morphological changes after light-stimulation.

(**A**) Image series of a living TlnKO cell co-expressing the light-insensitive Tln-AsLOV2I (magenta) and Zdk2-Tln (green) before and under light-stimulation. The irradiation area is indicated by the white dashed line. No morphological changes were observed. (**B**) Quantification of normalized cell area before, 25 s, and 75 s after the start of light stimulation (n=15; Paired sample Wilcoxon Signed Rank test). Scale bar indicates 10 µm. n.s. (not significant) $p \geq 0.05$.

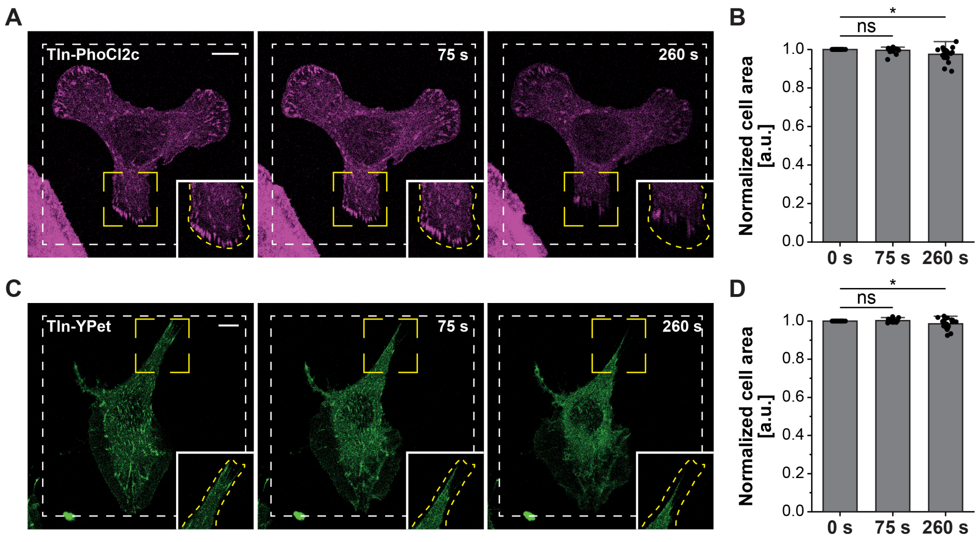

Fig. S5. Effect of light stimulation on cells expressing Tln-PhoCl2c.

(**A**) Representative image of a living TlnKO cell expressing Tln-PhoCl2c before and under light stimulation with 405 nm. In some instance, local retractions were observed. The irradiation area is indicated by the white dashed line, zoomed-in areas are outlined by yellow lines. (**B**) Quantification reveals a slight reduction in cell area after prolonged (260 s) exposure to 405 nm light (n=20; Paired sample Wilcoxon Signed Rank test). (**C**) Representative image of a living TlnKO cell expressing Tln-YPet before and under light stimulation with 405 nm. The occurrence of local retractions after prolonged light exposure indicates a non-specific, presumably phototoxic effect. Irradiation area is indicated by the white dashed line, zoomed-in areas are outlined by yellow lines. (**D**) Quantification of cell area in light-stimulated control cells expressing Tln-YPet indicates that the observed effects in Tln-PhoCl2c cells are unspecific (n=20; Paired sample Wilcoxon Signed Rank test). Scale bars indicates 10 µm. * $p < 0.05$, n.s. (not significant) $p \geq 0.05$.

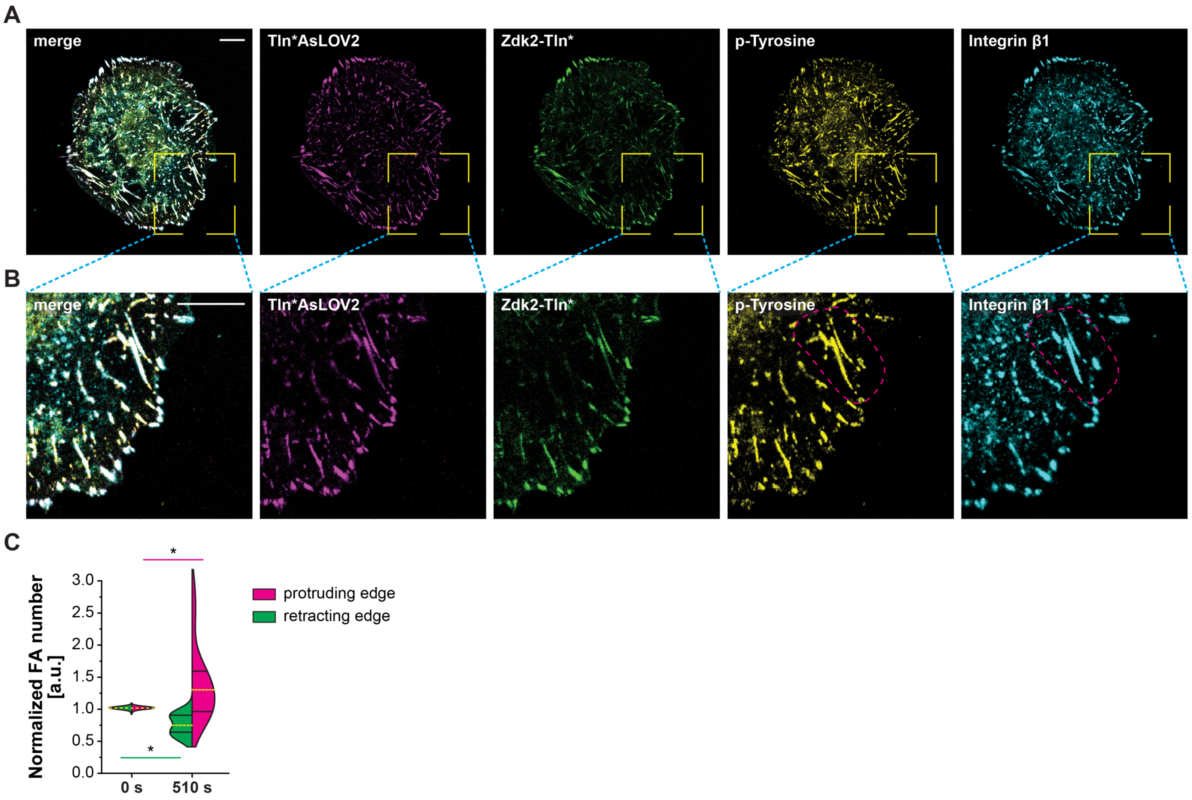

Fig. S6. Analysis of FA populations in Talin-1973-OM cells.

(**A** and **B**) Immunostaining of Talin-1973-OM cells with antibodies against phospho-tyrosine (p-tyrosine) and β1 integrin highlighting the enrichment of p-tyrosine in large peripheral focal adhesions (magenta, dotted). Zoomed-in regions are indicated by yellow dashed lines in (**A**). (**C**) Quantification of normalized FA number in retracting (green) versus protruding (magenta) edges of Talin-1973-OM cells. Note that the FA number tends to increase in protruding areas of the cell, while FA number decreases in retracting areas under light stimulation. Scale bar indicates 10 µm (**A**) and 5 µm (**B**). * $p < 0.05$.

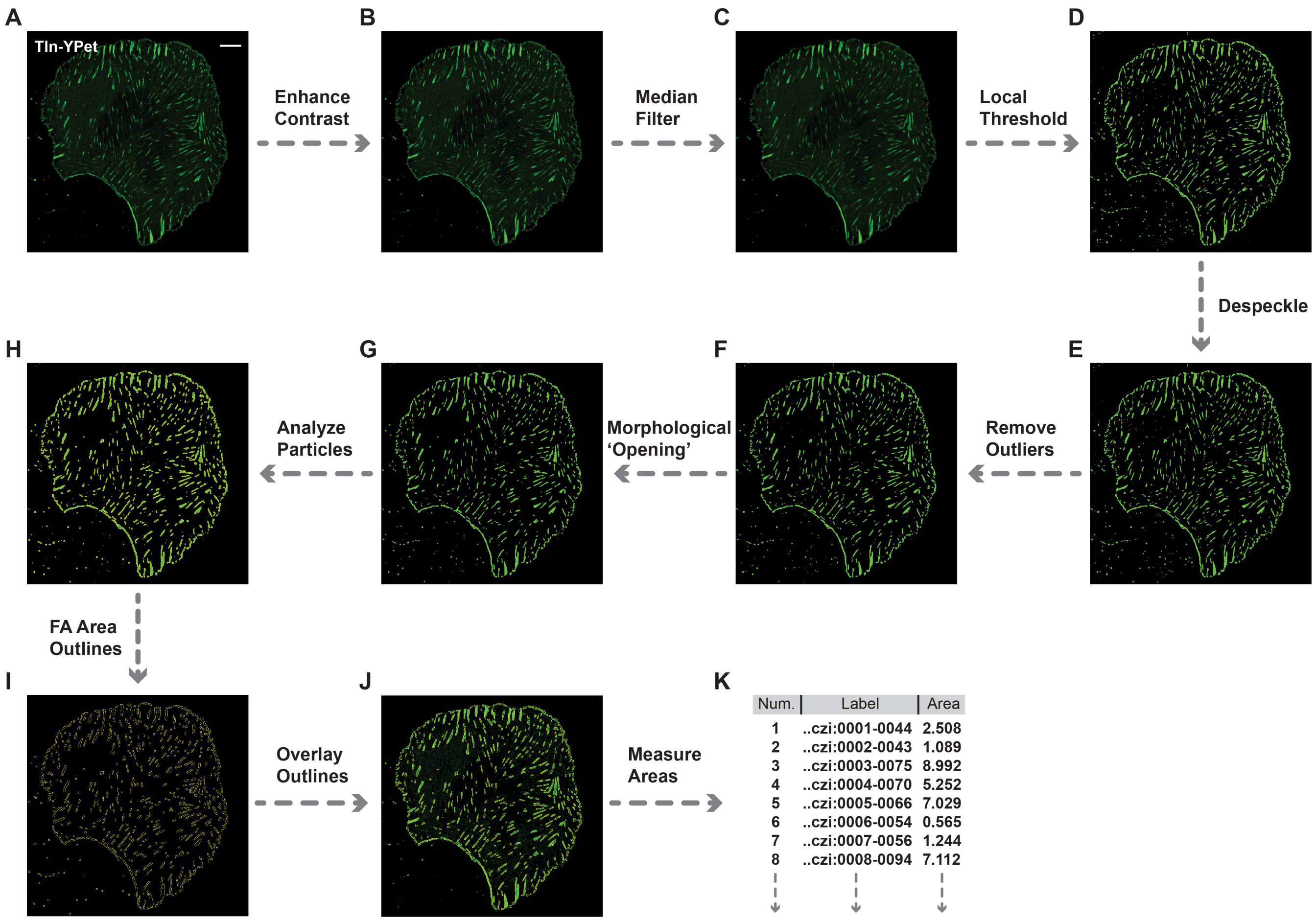

Fig. S7. Work flow to isolate and quantify focal adhesion areas.

(**A** to **K**) Schematic illustration of the work flow to filter a fluorescence FA image, using a Tln-YPet image (**A**) as an example. After a contrast enhancement (**B**) and median filtering (**C**), local thresholding was applied to isolate areas of interest (**D**). The derived mask was cleaned by 'despeckle' (**E**), removing outliers (**F**) and morphological opening (**G**) steps. Regions greater than 0.25 μm^2 were analysed (**H**) and their boundaries (**I**) laid over the original fluorescence image to verify that their position was within the cell area (**J**). Isolated FA regions were then used to calculate the FA area (**K**). Scale bar indicates 10 µm.

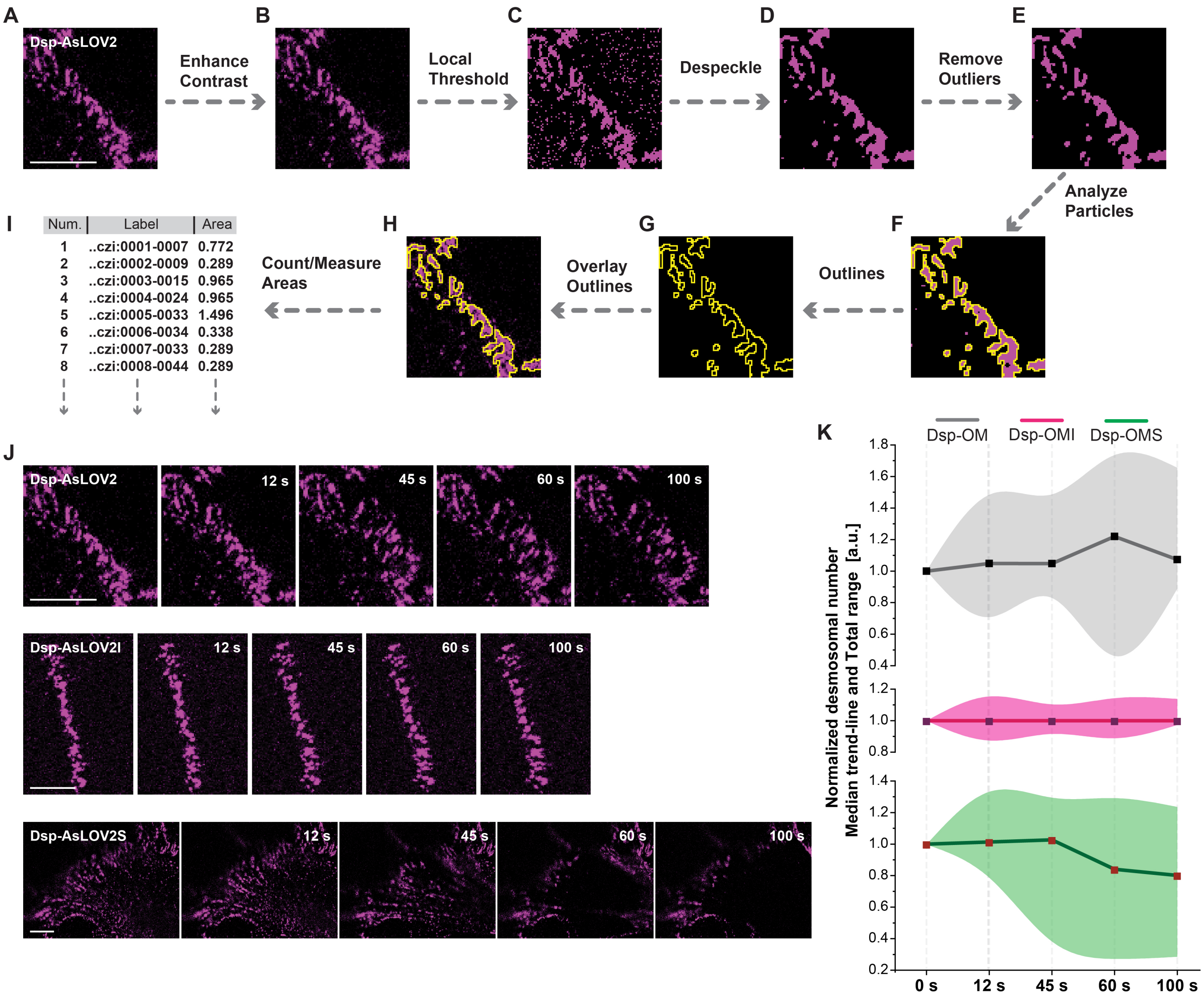

Fig. S8. Work flow to isolate and quantify amount of desmosomal clusters.

(**A** to **I**) Desmosomal area isolation routine showing an exemplary fluorescence image (**A**) of a Dsp-AsLOV2 expressing cell. Sequential contrast enhancement (**B**) and local thresholding (**C**) steps created a mask for ROIs that was further cleared by ‘despeckle’ (**D**) and removing outliers (**E**). Regions greater than 0.25 μm^2 were isolated and analysed (**F**), with their boundaries (**G**) laid over the original fluorescence image to confirm that their position was within the cell area (**H**). The number of isolated regions or ‘desmosomal clusters’ in the cell-cell junction was quantified (**I**). (**J**) Cells expressing Dsp-AsLOV2, the light-insensitive control Dsp-AsLOV2I, and the slow rebinding variant Dsp-AsLOV2S react differently to combined pulling and irradiation. (**K**) Quantification of desmosomal clusters over time revealed a slow dissociation for the Dsp-AsLOV2 construct (Dsp-OM, grey), no change for the light-insensitive Dsp-AsLOV2I (Dsp-OMI, magenta), and a rapid collapse in the Dsp-AsLOV2S mutant cells (Dsp-OMS, green). The graph shows the median value at distinct time points after light stimulation/pulling; the shaded area indicates the total range (TR) of the underlying data.

Movie S1. (separate file) Sudden loss of the talin-1 linkage induces disintegration of cell adhesion and cellular collapse.

Image series of a TlnKO cell expressing Tln-AsLOV2 (magenta) and Zdk2-Tln (green), exposed to 458 nm light (irradiated area indicated by dashed line). Sudden loss of the talin linkage triggers a cellular collapse, in which the YPet-labelled C-terminal fragment is dragged into the cell body while some of the mCherry-labelled N-terminal fragments remain at sites of initial adhesion. Scale bar indicates 10 µm.

Movie S2. (separate file) Light-insensitive control cell under light stimulation.

Image series of a TlnKO cell expressing the light-insensitive Tln-AsLOV2I (magenta) and Zdk2-Tln (green), exposed to 458 nm light (irradiated area indicated by dashed line). Cells do not respond to light stimulation. Scale bar indicates 10 µm.

Movie S3. (separate file) Cells expressing the slow-rebinding AsLOV2 variant are highly sensitive to light stimulation.

Image series of a TlnKO cell expressing the slow-rebinding variant Tln-AsLOV2S (magenta) and Zdk2-Tln (green), showing high sensitivity to 458 nm light stimulation (irradiated area indicated by dashed line). Scale bar indicates 10 µm.

Movie S4. (separate file) Absence of cellular collapse in light-stimulated cells expressing the fast-rebinding AsLOV2 variant.

Image series of a TlnKO cell expressing the fast-rebinding variant Tln-AsLOV2F (magenta) and Zdk2-Tln (green). Cells do not collapse under 458 nm light stimulation (irradiated area indicated by dashed line), presumably because the talin linkage is rapidly reconstituted. Scale bar indicates 10 µm.

Movie S5. (separate file) Tln-PhoCl–expressing cell under light stimulation.

Image series of a TlnKO cell expressing a talin-1 construct with PhoCl-mCherry inserted after aa 447 (Tln-PhoCl) (magenta). Light-stimulation at 405 nm (irradiated area indicated by dashed line) did not result in a cellular collapse or any significant change in cell area, even after prolonged stimulation. The movie shows the mCherry intensity. Scale bar indicates 10 µm.

Movie S6. (separate file) Tln-PhoCl2c–expressing cell under light stimulation.

Image series of a TlnKO cell expressing a talin-1 construct with PhoCl2c-mCherry inserted after aa 447 (Tln-PhoCl2c) (magenta). Light-stimulation at 405 nm (irradiated area indicated by dashed line) did not result in a cellular collapse. The movie shows the mCherry intensity. Scale bar indicates 10 µm.

Movie S7. (separate file) Abrogating talin-1 linkages in half of the cell.

Image series of a partially irradiated TlnKO cell expressing Tln-AsLOV2 (magenta) and Zdk2-Tln (green). FAs quickly disintegrate within the irradiated area (dashed line) but remain unaffected in the non-stimulated half of the cell. When the cell is allowed to recover in the absence of light stimulation (recovery times in minutes), FAs reform and the initial morphology is restored. Scale bar indicates 10 µm.

Movie S8. (separate file) Abrogating talin-1 linkages in individual FAs.

Image series of a TlnKO cell expressing Tln-AsLOV2 (magenta) and Zdk2-Tln (green). The stimulation of individual FAs with 458 nm light (dashed line) results in a specific collapse of the targeted adhesion area. Scale bar indicates 10 μm.

Movie S9. (separate file) Disengaging the C-terminal actin-binding domain of talin-1 with light.

Image series of a TlnKO cell expressing Tln*-AsLOV2 (magenta) and Zdk2-Tln* (green), stimulated with 458 nm light (dashed line). Large, peripheral FAs slowly retract but cells do not collapse under light-stimulation; newly forming FAs seem unaffected. Scale bar indicates 10 μm.

Movie S10. (separate file) No response in light-insensitive control cells.

Image series of a TlnKO cell expressing the light insensitive Tln*-AsLOV2I (magenta) and Zdk2-Tln* (green), stimulated with 458 nm light (dashed line). No change in FA stability and cellular area are observed even under prolonged stimulation. Scale bar indicates 10 μm.

Movie S11. (separate file) Disengaging the desmoplakin linkage.

Image series of a DspKO MEK expressing Dsp-AsLOV2 (magenta) and Zdk2-Dsp (green). Stimulation with 458 nm light (dashed line) does not affect the overall integrity of the cell-cell junction. Scale bar indicates 10 μm.

Movie S12. (separate file) Loss of cohesion in mechanically stressed intercellular junctions upon light stimulation.

Image series of a DspKO MEK expressing Dsp-AsLOV2 (magenta) and Zdk2-Dsp (green). Cells were exposed to mechanical stress by micropipette pulling and simultaneously stimulated with 458 nm light (dashed line). Junctions and the Dsp signals slowly separate leading to an increased number of detected desmosomal signals. Scale bar indicates 10 μm.

Movie S13. (separate file) Cells expressing the light-insensitive AsLOV2 variant resist mechanical stress under light stimulation.

Image series of a DspKO MEK expressing the light-insensitive Dsp-AsLOV2I (magenta) and Zdk2-Dsp (green). Cell-cell junctions remain associated when cells are stressed by mechanical pulling and simultaneously stimulated with 458 nm light (dashed line). Scale bar indicates 10 μm.

Movie S14. (separate file) Increased sensitivity of stressed intercellular junctions in cells expressing the slow-rebinding AsLOV2 variant.

Image series of a DspKO MEK expressing the slow-rebinding Dsp-AsLOV2S (magenta) and Zdk2-Dsp (green). Rapid dissociation and junctional collapse were observed upon application of mechanical stress and simultaneous stimulation with 458 nm light (dash line). The rapid loss of cell-cell adhesion causes a loss of desmosomal signal in the analysed area and thus a decrease in detected desmosomal numbers. Scale bar indicates 10 μm.

Data S1. (separate file)

Source data for graphs, plots and curves in various insets.
